# 2885. Increased incidence of fluconazole-resistant *Candida parapsilosis* bloodstream infections in Atlanta, Georgia 2021

**DOI:** 10.1093/ofid/ofad500.162

**Published:** 2023-11-27

**Authors:** Lucy S Witt, Elizabeth Misas, Monica M Farley, Stepy Thomas, Emily N Jenkins, Scott Fridkin, Nancy A Chow, Shawn R Lockhart, Meghan Lyman

**Affiliations:** Emory University, Atlanta, GA; ORISE-CDC, Atlanta, Georgia; Emory University School of Medicine, Division of Infectious Diseases, Atlanta, Georgia; Emory University, Atlanta, GA; ASRT, Inc.; Centers for Disease Control and Prevention, Atlanta, Georgia; Georgia Emerging Infections Program, Decatur, GA; Emory University School of Medicine, Atlanta, GA, Atlanta, Georgia; CDC, Atlanta, GA; Centers for Disease Control and Prevention, Atlanta, Georgia; Centers for Disease Control and Prevention, Atlanta, Georgia

## Abstract

**Background:**

Since 2020, outbreaks of fluconazole-resistant *Candida parapsilosis* (FR-CP) infections have proliferated around the world. An increase in FR-CP bloodstream infections (BSI) in the Atlanta metropolitan area in 2021 was identified during routine surveillance through the Georgia Emerging Infections Program supported by the Centers for Disease Control and Prevention (CDC). We describe the genomic relatedness and epidemiologic associations between resistant and susceptible isolates.

**Methods:**

Active laboratory- and population-based surveillance for candidemia was conducted in Atlanta during 2018-2021. Fluconazole susceptibility testing of CP isolates was performed at the CDC in accordance with CLSI breakpoints. Whole-genome sequencing was completed on 82% of all viable CP isolates from 2021. Clinical and epidemiologic data was abstracted from medical records. Bivariate analysis was performed using Wilcoxon-rank sum and chi-squared (or Fisher’s) test as appropriate.

**Results:**

A total of 60 confirmed cases of CP BSI were characterized in 2021. Incidence of CP BSIs increased along with the proportion of FR-CP isolates in 2020-21(Figure 1). FR-CP isolates were genetically closely related (0-39 SNPs), and all contained the Y132F substitution in the *Erg11* gene (Figure 2). FR-CP BSI (n = 19) were more common in patients who were admitted to a long-term acute care hospital (LTACH) in the last 90 days (p= < 0.01) and those who were mechanically ventilated in the 30 days prior (p=0.02). FR-CP was less common in patients who were in a short-term acute care hospital at the time of diagnosis (p=0.01) or who had a malignancy (p=0.03) (Figure 3). Patients with FR-CP isolates interacted most frequently with a unique set of healthcare facilities as compared to susceptible isolates (data not shown).

**Figure 1.**
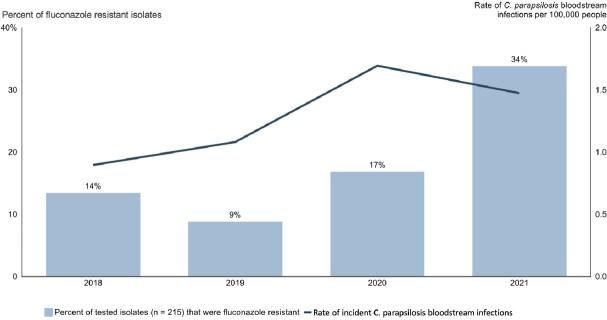
Rate of incident Candida parapsilosis bloodstream infections by year with percent of fluconazole-resistant isolates

**Figure 2.**
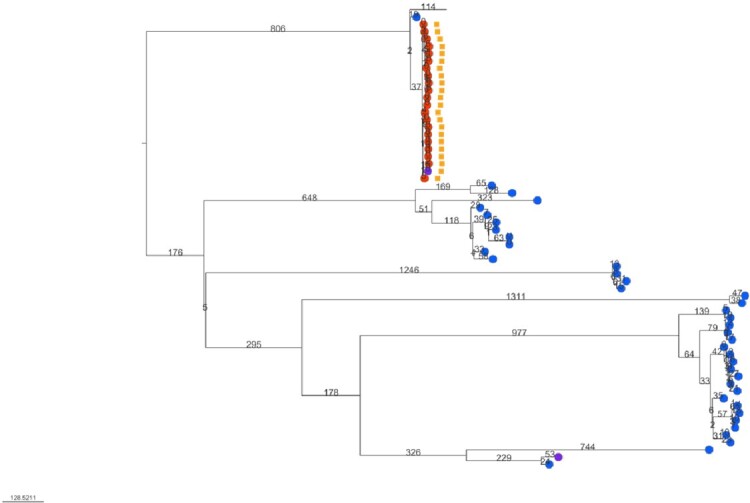
Neighbor-joining phylogeny of Candida parapsilosis, isolates from 2021.

Branch length indicates the number of SNPs and taxa nodes’ color indicates fluconazole-susceptible/resistant phenotype; no-circle taxa indicate the reference isolate. External orange squares flag isolates with the mutation Y132F in the Erg11 gene.

**Figure 3.**
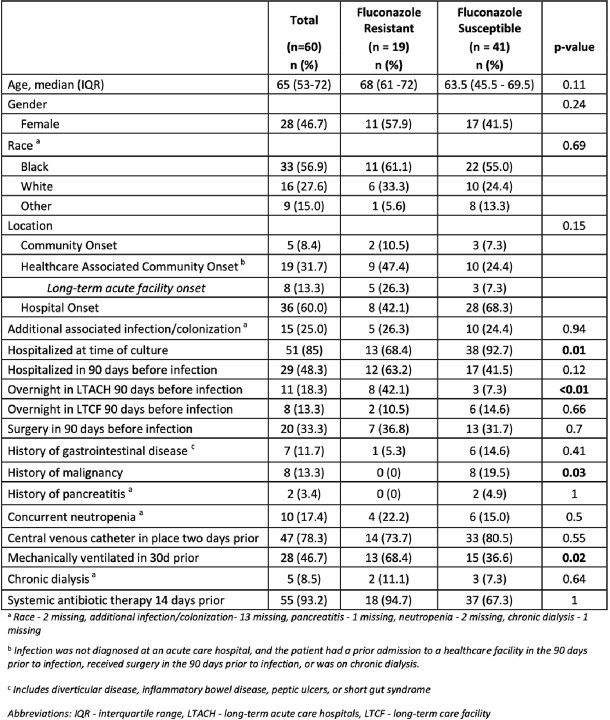
Characteristics of patients with Candida parapsilosis infections in Atlanta, GA 2021 by fluconazole susceptibility

**Conclusion:**

Overall rates of *Candida parapsilosis* BSIs in Atlanta increased in 2020 during the COVID-19 pandemic and highly related isolates of FR-CP emerged in 2021, associated with significant recent past exposure to LTACHs and mechanical ventilation. Network analysis is underway to further evaluate patterns of transmission that may inform infection prevention efforts.

**Disclosures:**

**All Authors**: No reported disclosures

